# Neurogenic Pulmonary Edema Associated with Hyponatremia, Primary Polydipsia, and Cannabis Use: A Case Report

**DOI:** 10.5811/cpcem.6562

**Published:** 2024-06-03

**Authors:** Christian Treat, Nicholas Ulloa, Alyssa Kettler, David Lawrence

**Affiliations:** Stony Brook University, Department of Emergency Medicine, Stony Brook, New York

**Keywords:** *neurogenic pulmonary edema*, *hyponatremia*, *primary polydipsia*, *cannabis intoxication*, *case report*

## Abstract

**Introduction:**

Neurogenic pulmonary edema is a rare and potentially life-threatening condition that can present as severe pulmonary edema after significant neurologic insults. This is the first documented instance that shows a plausible causal link between cannabis consumption, psychogenic polydipsia, and the subsequent development of neurogenic pulmonary edema associated with status epilepticus secondary to acute hyponatremia.

**Case Report:**

We report a case of a 34-year-old female who presented to the emergency department altered and postictal after a witnessed new-onset seizure. She developed significant respiratory distress that required intubation. Her sodium was 121 millimoles per liter (mmol/L), from 137 mmol/L 36 hours prior on routine outpatient labs. Further history revealed excessive water ingestion after eating a cannabis edible prior to the seizure.

**Conclusion:**

This case highlights the importance of recognizing neurogenic pulmonary edema in connection with psychogenic polydipsia, severe hyponatremia, and status epilepticus subsequent to cannabis consumption.

Population Health Research Capsule What do we already know about this clinical entity?

*Neurogenic pulmonary edema (NPE) is a rare, life-threatening complication arising from neurological insults such as seizures and intracranial hemorrhage.*

What makes this presentation of disease reportable?

*This report highlights a novel case of psychogenic polydipsia from cannabis use leading to NPE with status epilepticus due to hyponatremia.*

What is the major learning point?

*Cannabis use may lead to psychogenic polydipsia, symptomatic hyponatremia, and potentially induce neurologic sequelae including seizures and NPE.*

How might this improve emergency medicine practice?

*Patterns of increasing cannabis use in the United States show the need to consider lesser known complications in patients with cannabis-related ED visits.*

## INTRODUCTION

Neurogenic pulmonary edema (NPE) is a rare and potentially life-threatening condition that can occur as a complication of neurologic insults such as seizures, traumatic brain injury, and intracranial hemorrhage.^1^ It is believed to result from a sudden increase in sympathetic tone leading to increased pulmonary capillary hydrostatic pressure and increased pulmonary vascular permeability.^2^ ^,^ ^3^ Neurogenic pulmonary edema is often associated with significant morbidity and mortality, and early recognition and management are crucial for favorable outcomes. In this case report, we illustrate a plausible causal link between cannabis consumption, psychogenic polydipsia, and the subsequent development of NPE associated with status epilepticus secondary to acute hyponatremia.

## CASE REPORT

A 34-year-old female with a medical history of well-controlled HIV, depression, and anxiety presented to the emergency department (ED) with new-onset seizures. According to her partner, she had consumed an edible cannabis cookie earlier in the day and then drank approximately eight 16.9-ounce bottles of water over the course of one hour in response to her severe thirst. Emergency medical services were called because she had a witnessed, generalized tonic-clonic seizure that lasted approximately five minutes. Prehospital vital signs were notable for peripheral oxygen saturation (SpO_2_) of 69% on room air and a fingerstick glucose of 254 milligrams per deciliter (mg/dL) (normal range 70–99 mg/dL).

On arrival to the ED, the patient’s heart rate was 104 beats per minute, blood pressure 117/71 millimeters of mercury (mm Hg), respiratory rate of 39 breaths per minute, and SpO_2_ 69% on room air. A non-rebreather mask was placed with improvement of SpO_2_ to 90%. She was combative and altered, unable to follow commands to not remove the non-rebreather mask, and demanding water from ED staff. Due to excessive agitation, 50 mg of intravenous (IV) ketamine was given to the patient to allow us to obtain an emergent computed tomography (CT) head without contrast, CT angiogram head and neck, and CT chest without contrast. Following CT imaging, she had another seizure and her SpO_2_ in the low 80% range despite non-rebreather mask. In the setting of her refractory hypoxia, altered mental status, and seizures, the decision was made to intubate. We used a delayed sequence intubation to preoxygenate the patient by first administering 100 mg of IV ketamine. This improved SpO_2_ to 96%. Then we proceeded with paralysis with 100 mg of rocuronium and intubated successfully without episodes of hypoxia. Post-intubation, we administered propofol at a rate of 20 micrograms (mcg)/kg/minute.

Laboratory evaluation of the patient revealed marked hyponatremia of 121 millimoles per liter (mmol/L), compared to her baseline level of 137 mmol/L obtained 36 hours prior during a routine outpatient visit (normal range 135–146 mmol/L). Two ampules (100 mL) of 8.4% sodium bicarbonate were administered to the patient for acute symptomatic hyponatremia. Acetaminophen, salicylate, and ethanol levels were all negative. A urine toxicology screen was positive for cannabis, and an expanded urine toxicology panel was sent to an external laboratory. Emergent CT imaging showed normal findings on non-contrast head and angiography of the head and neck, and a CT perfusion study demonstrated no signs of acute infarction. The non-contrast CT chest revealed multifocal airspace and diffuse bilateral ground-glass opacities, with no evidence of effusion or pneumothorax.

Arterial blood gas analysis revealed the following results after intubation: pH 7.20 (normal range 7.35–7.45), partial pressure of carbon dioxide (PaCO_2_) 59 mm Hg (35 to 45 mm Hg), partial pressure of oxygen (PaO_2_) 83 mm Hg (normal range 80–100 mm Hg), and bicarbonate 23 mmol/L (22–26 mmol/L). The patient’s ventilation status necessitated high levels of positive end-expiratory pressure (PEEP) and fraction of inspired oxygen (FiO_2_), 16 centimeters H_2_O and 100%, respectively. She was admitted to the intensive care unit for acute hyponatremia and hypoxemic respiratory failure. After receiving two ampules of sodium bicarbonate in the ED, her sodium levels increased from an initial level of 121 mmol/L to 123 mmol/L after four hours. Subsequently, she was administered two doses of 100 mL of 3% saline, resulting in a repeat sodium level of 131 mmol/L after 13 hours from initial sodium level. Finally, her sodium reached 135 mmol/L after 29 hours from initial level. Her expanded drug toxicology panel that was sent to an outside laboratory reported that her urine was positive for >500 mcg/mL 11-nor-9-carboxy-tetrahydrocannabinol (the psychoactive compound found in cannabis) and negative for 3,4-methylenedioxymethamphetamine (MDMA), amphetamines, barbiturates, benzodiazepine, cocaine, methadone, opiates, phencyclidine, and propoxyphene.

The patient’s ventilatory requirements were weaned to FiO_2_ 40%, PEEP 10 cm H_2_O, with pH 7.35, PaCO_2_ 37 mm Hg, and PaO_2_ 155 mm Hg, and she was extubated to four liters nasal cannula14 hours after intubation and transferred to the general medical floors on hospital day three.

An echocardiogram performed showed normal cardiac parameters with an ejection fraction of 71% and normal valvular structures. An electrocardiogram showed sinus rhythm, short PR interval, narrow QRS, and normal QTc. Telemetry was without evidence of any ventricular arrhythmias. Follow-up chest radiographs (CXR) showed resolving pulmonary edema.

Intravenous ceftriaxone and azithromycin were continued for her hospital course. The patient was afebrile, had no leukocytosis, and reported no respiratory issues post-extubation. The infectious workup also included a sputum culture with normal flora, negative urinary legionella and pneumococcal antigens, negative blood cultures, and negative urine cultures. Additionally, a CD4 level drawn two days prior to admission was 752 cells per microliter, HIV viral load was undetectable, and a respiratory pathogen panel, including testing for respiratory syncytial virus, influenza virus, and SARS-CoV-2, was negative. The patient underwent 24-hour video electroencephalogram monitoring, which revealed no seizure activity and she returned to normal neurological status by hospital day one after sodium correction.

On social work evaluation, she was adherent to her mental healthcare treatment regimen, regularly attended therapy, and used escitalopram for depression and anxiety. She had no documented psychiatric hospitalizations, suicidal or homicidal ideation, or hallucinations in the prior year. She was discharged to home on hospital day six. She reported no acute concerns at her subsequent primary care and nephrology follow-up visits, seven and 22 days later, respectively.

## DISCUSSION

This case shows the development of NPE from severe acute hyponatremia caused by psychogenic polydipsia related to cannabis use. To our knowledge, this specific acute complication of cannabis has not been extensively reported. Preliminary data has shown the association between chronic cannabis use and hyponatremia. In rat models, chronic marijuana administration reduced serum sodium in rats over a 12-week period, likely via inhibitor effects on sodium channels.^4^ Another study used spectrophotometry to estimate serum sodium levels in a sample of regular marijuana smokers and non-marijuana smoking controls and found significantly decreased mean sodium levels of 119 ± 26 mmol/L compared to mean sodium levels of 140 ± 6 mmol/L.^5^ Taken together, this evidence indicates marijuana can disrupt sodium balance, which emergency physicians should recognize as an important possible complication of cannabis use.

Any acute central nervous system event that abruptly triggers a sympathetic discharge can potentially trigger NPE. Multiple references link status epilepticus to noncardiogenic pulmonary edema, emphasizing the relevance of considering NPE in the context of seizure-related complications. For instance, one report described a stronger association between seizures lasting greater than 200 seconds and the development of pulmonary edema on CXR.^6^

Proposed criteria for the diagnosis of NPE include the following: bilateral opacities, PaO_2_/FiO_2_ ratio <200; no evidence of left arterial hypertension, presence of neurologic injury, and absence of other causes of acute respiratory distress syndrome.^7^ Our patient, who presented with first-time seizures, had bilateral opacities and met criteria for severe acute respiratory distress syndrome (ARDS) with a PaO_2_/FiO_2_ of 83. The echocardiogram findings, including normal left ventricular size, wall thickness, global systolic function, absence of hypertrophy, and no signs of impaired diastolic function or left atrial enlargement, collectively suggested no evidence of left arterial hypertension in the patient.

Intriguingly, Ayus-Arieff syndrome is a condition characterized by hyponatremia-induced cerebral edema leading to the development of pulmonary edema and has been documented in case reports, particularly in contexts involving MDMA use and in marathon runners.^8^ ^,^ ^9^ No reports currently link it to cannabis consumption. The syndrome became a significant consideration for our patient, who experienced new-onset seizures in the context of acute hyponatremia, attributable to polydipsia secondary to cannabis intoxication. Subsequently, she required intubation due to acute hypoxemic respiratory failure associated with ARDS. Notably, a thorough workup revealed normal cardiological and infectious parameters with a rapid return to clinical baseline following correction of sodium.

Effective management of hyponatremia requires a tailored approach based on underlying causes and severity. Hyponatremia stems from diverse causes, including severe renal failure, excessive water intake, hypovolemic factors such as gastrointestinal losses or diuretics, euvolemic conditions such as adrenal insufficiency and syndrome of inappropriate antidiuretic hormone (SIADH), and edematous states such as heart failure or cirrhosis. Patients with acute hyponatremia exhibit serum sodium levels below 135 mmol/L within 48 hours, while chronic hyponatremia develops over more than 48 hours or is assumed in the absence of a known baseline. The management of hyponatremia is critically influenced by its chronicity and severity. Immediate concerns in symptomatic hyponatremia involve seizures, cerebral edema, and brain herniation. In chronic cases, the risk lies in the potential for rapid correction. Although osmotic demyelination is rare if the initial sodium level exceeds 120 mmol/L, it becomes a concern with a high rate of sodium rise, typically surpassing 8 mmol/L in a 24-hour period.^10^

The patient’s acute hyponatremia appeared to be linked to psychogenic polydipsia given the historical context, likely due to excessive water intake in the setting of cannabis intoxication. Given her history of depression and anxiety, an alternative diagnosis could be SIADH, which is known to be associated with antidepressants.^11^ This syndrome tends to have a more insidious onset, making this diagnosis less likely as her sodium was within normal range the day prior to hospitalization.

In terms of management, patients who develop severe symptoms such as seizures or coma, should be treated promptly and aggressively with hypertonic 3% saline. Of note, multiple studies confirm that 3% saline is safe to administer through a peripheral line.^12^ ^–^ ^14^ Therefore, attempting central access is unnecessary and should not delay treatment. Depending on the ED, 3% saline may require the pharmacy to prepare, which will further delay treatment. In those circumstances, ampules of sodium bicarbonate are a resourceful alternative for the treatment of severe hyponatremia. For example, one ampule of sodium bicarbonate has the same osmolarity of 5.8% saline, essentially twice the osmolarity of 3% saline.^15^ Therefore, 50 mL of sodium bicarbonate, or one ampule, is roughly equivalent to 100 mL of 3% saline. For our patient, we administered 100 mL, or two ampules, of sodium bicarbonate because it was the most rapid method to treat her severe hyponatremia. This increased her sodium to 123 mmol/L, which was within our goal.

## CONCLUSION

This case underscores the rare occurrence of neurogenic pulmonary edema linked to psychogenic polydipsia, severe hyponatremia, and status epilepticus following cannabis consumption. The significance of this potential association with cannabis is heightened against the backdrop of the escalating consumption, decriminalization, and legalization trends observed in the United States. This not only underscores the clinical complexity of the case but also emphasizes the urgent need for thorough investigation and awareness as cannabis usage patterns evolve.
